# Effect of C242T Polymorphism in the Gene Encoding the NAD(P)H Oxidase p22^phox^ Subunit and Aerobic Fitness Levels on Redox State Biomarkers and DNA Damage Responses to Exhaustive Exercise: A Randomized Trial

**DOI:** 10.3390/ijerph17124215

**Published:** 2020-06-12

**Authors:** Su-Youn Cho, Wi-Young So, Hee-Tae Roh

**Affiliations:** 1 Exercise Physiology Laboratory, Department of Physical Education, Yonsei University, Seoul 03722, Korea; csy@yonsei.ac.kr; 2 Sports and Health Care Major, College of Humanities and Arts, Korea National University of Transportation, Chungju-si 27469, Korea; wowso@ut.ac.kr; 3 Department of Physical Education, College of Arts and Physical Education, Dong-A University, Busan 49315, Korea

**Keywords:** *CYBA* gene, oxidative stress, maximum oxygen uptake

## Abstract

NAD(P)H oxidases (NOXs) constitute a principal source of cellular reactive oxygen species (ROS) and contribute to exercise-induced ROS production in the skeletal muscle. Here, we aimed to investigate the effect of single-bout exhaustive exercise on redox state biomarkers and oxidative DNA damage based on the C242T polymorphism in the gene encoding NOXs subunit p22^phox^ (*CYBA*) and aerobic fitness levels. We enrolled 220 healthy adults in their 20s (men, *n* = 110; women, *n* = 110), who were divided into CC genotype and T allele groups through the analysis of the *CYBA* C242T polymorphism. Furthermore, maximum oxygen uptake (VO_2_max) was evaluated to divide subjects into high fitness (HF; 70th percentile for aerobic fitness) and mid-range fitness (MF; 40–60th percentile for aerobic fitness) groups, with a total of 32 subjects assigned to four groups (eight subjects per group): CC genotype and HF group (CC + HF), CC genotype and MF group (CC + MF), T allele and HF group (T + HF), and T allele and MF group (T + MF). All subjects performed treadmill running exercise at 85% of VO_2_max until exhaustion. Plasma lactate, malondialdehyde (MDA), superoxide dismutase (SOD), and lymphocyte DNA damage (tail DNA percentage [TD], tail length [TL], and the tail moment [TM]) were measured in the blood samples obtained immediately before (IBE), immediately after (IAE), and 30 min after exercise (30 MAE). Plasma lactate levels, SOD activities, and lymphocyte DNA damage markers (TD, TL, and TM) were significantly increased at IAE than that at IBE and significantly decreased at 30 MAE (*p* < 0.05). All groups displayed increased plasma MDA levels at IAE rather than at IBE, with CC + MF being significantly higher than T + HF (*p* < 0.05); only the CC + HF and T + HF groups exhibited a significant reduction at 30 MAE (*p* < 0.05). Moreover, TL at IAE was significantly higher in the CC + MF group than in the T + HF group (*p* < 0.05), and significantly higher in the CC + MF and CC + HF groups than in the T + HF group at 30 MAE (*p* < 0.05). TM was significantly higher in the T + MF than in the T + HF group at IAE (*p* < 0.05) and that of CC + MF was significantly higher than CC + HF and T + HF values at IAE and 30 MAE (*p* < 0.05). These results suggest that single-bout exhaustive exercise could induce peripheral fatigue and the accumulation of temporary redox imbalance and oxidative DNA damage. Moreover, high aerobic fitness levels combined with the T allele may protect against exercise-induced redox imbalance and DNA damage.

## 1. Introduction

Oxidative stress (OS) is induced when the body’s oxidant and antioxidant systems are unbalanced, as oxidation reactions predominate in the redox state [1]. Regular exercise training, which promotes antioxidant capacity, exerts antioxidant effects that can alleviate OS levels [2]. Alternatively, high-intensity exhaustive exercise requiring 10-fold oxygen supply and adenosine triphosphate (ATP) induces excessive reactive oxygen species (ROS) and free radical production, resulting in a rapid increase of the body’s OS-level [3,4]. Excessive ROS lowers exercise performance by inducing fatigue, owing to reduced calcium sensitivity during exercise, potentially resulting in inflammatory reactions and muscle damage [5,6,7]. Furthermore, it produces lipid peroxide through chain reactions with other fat components and can induce oxidative damage to cellular components, including lipids, proteins, and/or DNA [7,8,9]. Mitochondria, nicotinamide adenine dinucleotide phosphate oxidases (NOXs), phospholipase A2 (PLA2)-dependent processes, and xanthine oxidase are reportedly involved in ROS production [9]. Among these, NOXs constitute a principal source of cellular ROS and contribute to exercise-induced ROS production in skeletal muscles [9,10].

NOX is a multi-enzyme complex comprising the subunit proteins gp91^phox^, p22^phox^, p47^phox^, p67^phox^, and p40^phox^ [10,11]. Although p22^phox^ is expressed in non-phagocytic cells such as fibroblasts, endothelial cells and vascular smooth muscle cells, it is primarily expressed in phagocytic cells and is associated with superoxide production, which is decreased upon p22^phox^ suppression [12,13]. p22^phox^ is a ubiquitous protein and is encoded by the cytochrome blight chain (*CYBA*) gene, containing six exons, located on chromosome 16q24, for which over 177 polymorphisms have been reported [14,15]. Among these, the three genotypes of the C242T polymorphism (CC, CT, and TT) [16,17] are reportedly associated with differences in whole-body OS induction [18,19,20]. For example, Guzik et al. [18] examined NOX activity in 110 patients with coronary artery disease risk factors by obtaining samples from the saphenous vein. They reported that vascular NOX activity significantly decreases in subjects harboring the T allele and suggested that the T allele of C242T is associated with lower superoxide production [18]. Hashad et al. [19] examined the oxidized low-density lipoprotein (ox-LDL) levels, a blood OS marker, in the serum of 104 patients with acute myocardial infarction. They reported that patients with the CT genotype presented significantly lower ox-LDL levels than those harboring the CC genotype. Moreover, Meijles et al. [20] reported that the saphenous vein, harboring the TT allele, exhibited significantly lower superoxide generation upon high-glucose treatment. However, most studies have evaluated the effects of this polymorphism regarding the pathophysiological aspects of cardiovascular diseases; thus, the role of these variants in healthy subjects is unclear.

High aerobic fitness potentially promotes endogenous antioxidant enzyme activation and is associated with high-intensity exercise-induced OS and DNA damage. Repka and Hayward [21] reported significant inverse correlations between peak oxygen uptake (VO_2_peak) and plasma 8-hydroxy-2′-deoxyguanosine (8-OHdG) levels. Moreover, Djordjevic et al. [22] reported that superoxide dismutase (SOD) activity was significantly higher in athletes than in non-athletes. In addition, Bachi et al. [23] reported that athletes with high VO_2_max displayed significantly lower ox-LDL levels after a marathon. Moreno-Villanueva et al. [24] recently reported that untrained subjects (VO_2_max < 45 mL/kg/min) exhibited increased DNA strand breaks in lymphocytes after one-bout exercise compared to trained (VO_2_max > 55 mL/kg/min) subjects.

Furthermore, the *CYBA* C242T polymorphism is reportedly associated with the exercise-induced OS, as *CYBA* requires the activation of oxidative enzymes in smooth muscle cells and is expressed in the coronary artery. However, studies investigating the effects of this polymorphism on OS levels regarding aerobic fitness status, which constitutes an important factor affecting body oxidant/antioxidant balance, are limited because most of them have been conducted under resting conditions with patients presenting pathologies including cardiovascular disease. Thus, in this study, we aimed to examine the exhaustive exercise-induced change in redox state biomarkers and oxidative DNA damage following the C242T polymorphism in the gene encoding p22^phox^ by evaluating healthy subjects at different aerobic fitness levels.

## 2. Methods

### 2.1. Participants

We enrolled 220 healthy adults in their 20s (110 men and 110 women), who voluntarily participated in the study following its advertisement at the Exercise Physiology Laboratory, Department of Physical Education, Yonsei University, Seoul, Korea. The inclusion criteria were as follows: (1) normal body composition [body mass index (BMI) < 25 kg/m^2^]; (2) no chronic health-related problems; (3) no history of cardiovascular, respiratory, or metabolic disease; (4) no smoking; and (5) no antioxidant intake in the past 3 months. *CYBA* C242T polymorphism was analyzed in all recruited adults, among which 89, 19, and 2 men and 91, 17, and 2 women harbored the CC, CT, and TT genotype, respectively. The genotypic and allelic frequencies of the C242T polymorphism herein are indicated in Table 1; subjects with the CC genotype were assigned to the CC group, and those harboring the T allele (CT and TT genotype) were assigned to the T group.

This study’s design was based on: (1) high fitness with the CC genotype (CC + HF), (2) mid-range fitness with the CC genotype (CC + MF), (3) high fitness with the T allele (T + HF), or (4) mid-range fitness with the T allele (T + MF) in four parallel groups, which were balanced and randomly allocated. The number of subjects necessary to ensure statistical significance and adequately address the study objective was determined using G*power version 3.1.9 (Heinrich-Heine-University, Düsseldorf, Germany). We then analyzed the minimum sample size at the effect size f = 0.40, α value = 0.05, and power (1 − β) = 0.90, and four groups of six individuals with each of the four genotypes of the C242T polymorphism and aerobic fitness levels. Considering potential drop-out, the VO_2_max test was conducted to include eight subjects for each group; however, there was no drop-out; therefore finally, eight subjects were included in each group (32 subjects). Subjects were then classified according to aerobic fitness level. Those corresponding to >70th percentile (men: VO_2_max ≥ 49.0 mL/kg/min; women: VO_2_max ≥ 41.0 mL/kg/min) for adults in their 20s (20–29-year-old), following the American College of Sports Medicine guidelines [25], were assigned to the HF group and subjects at the 40–60th percentile (men: VO_2_max = 42.6–47.4 mL/kg/min; women: VO_2_max = 36.2–39.4 mL/kg/min) to the mid-range fitness (MF) group. This study recruited 220 healthy students (110 males and 110 females) in their 20s and evaluated the *CYBA* C242T polymorphism. Results showed that 180 students had the CC genotype, and 40 students had the T allele (CT and TT genotype). Students with the CC genotype or T allele were randomly selected for VO_2_max testing; they were divided into the following groups (eight subjects per group): CC + HF, high fitness with the CC genotype; CC + MF, mid-range fitness with the CC genotype; T + HF, high fitness with the T allele; and CC + MF, mid-range fitness with the T allele. Of the original 220 subjects, 60 subjects completed VO_2_max testing, and 32 subjects were selected for the study groups. Moreover, we recruited the subjects and performed measurements for three months. The study objectives, methods, and protocols and data collection items were described to all subjects in detail, and the subjects signed a consent form, including various items such as voluntary cessation of study participation. The study protocol was approved by the ethics committee of the Korea Institute of Sport Science (KISS-06-A04004), and the study conformed to the standards set by the latest revision of the Declaration of Helsinki. However, this study was not a clinical trial. For that reason, we did not register the study and cannot provide the registration information. The characteristics of the subjects are shown in Table 2. No significant difference was detected for any variables between groups except for VO_2_max.

### 2.2. Basic Testing

Basic testing of subjects included VO_2_max, height, and body composition. VO_2_max testing was conducted using a gas analyzer (Meta Max 3B, Cortex, Leipzig, Germany) with the breath method on a treadmill (Quinton Q65, Hillrom, Chicago, IL, USA), as described by Bruce et al. [26]. VO_2_max was utilized if the heart rate was under 10% of the predicted heart rate and respiratory exchange ratio exceeded 1.10. Height and body composition were determined using a stadiometer (SECA213; SECA, Hamburg, Germany) and a bio-impedance analysis device (Inbody220; Biospace, Seoul, Korea), respectively. The subjects donned short-sleeves and pants and removed all metals before body composition measurement. In addition, the intake of any beverage or food from 4 h before the measurement was restricted, and the subject was asked to urinate to minimize the effect of body water. The safety of the participants was considered regarding the measurement of body composition following the Book of the American College of Sports Medicine’s (ACSM) Body Composition Assessment [27].

### 2.3. Exhaustive Exercise Test Protocol

Exhaustive exercise testing included treadmill running at 85% of VO_2_max, which was measured using the VO_2_max test, until the subject approached exhaustion and could not continue running. VO_2_max was measured using the previously published protocol by Bruce et al. [26], while the subject was wearing a gas analyzer. Exercise intensity was controlled by maintaining a steady state of VO_2_ by adjusting the speed and grade of the treadmill when the subject approached 85% of the VO_2_max as purposed.

### 2.4. Blood Sampling and Analyses

Blood was sampled from an antecubital vein of each subject using a 21-gauge needle; 10 mL blood was extracted into a plastic ethylene diamine tetraacetate (EDTA) tube (Becton Dickinson, Franklin Lakes, NJ, USA) immediately before (IBE), immediately after (IAE), and 30 min after exercise (30 MAE). Sampled blood was centrifuged (HA-12; HANIL Science Industrial, Incheon, Korea) at 3000 rpm for 15 min and stored at −80 °C until the analysis.

#### 2.4.1. C242T Polymorphism Analysis

Genomic DNA extraction was conducted using a DNA isolation kit (Gentra Genomic DNA purification kit; Minneapolis, MN, USA) following the manufacturer’s protocol. C242T genotype analysis was conducted using the SNP-ITTM method, which incorporates the single primer extension technology (SNPstream 25 KTM System; Orchid Biosystems, Princeton, NJ, USA). For polymerase chain reaction (PCR) amplification, we used one phosphorthiolated and one regular PCR primer: forward, 5′-AAAGGAGTCCCGAGTGGG-3′; reverse, 5′-AACATAGTAATTCCTGGTAAAGGG-3′. PCR conditions were as follows: 30 cycles at 95 °C for 30 s, 60 °C for 1 min, and 72 °C for 1 min; followed by denaturation for 1 min at 95 °C; and amplification for 7 min at 72 °C. Furthermore, as a single-stranded PCR template, 5′d-AACAGCTTCACCACGGCGGTCATGT-3′ was placed on a 96-well plate containing attached SNP-ITTM. Mixed nucleotides were classified using a series of colorimetric assays incorporating streptavidin-HRP and anti-FITC-AP. Yellow and/or blue color formation was analyzed using an ELISA reader, and the final genotype was determined using the QCReview^™^ software (Orchid Biosciences, Princeton, NJ, USA).

#### 2.4.2. Plasma Lactate, Malondialdehyde (MDA), and Superoxide Dismutase (SOD) Analyses

The absorbance of the samples was determined at 555 nm using an Ektachem DT 60 clinical chemistry analyzer (Eastman Kodak, Rochester, NY, USA) to analyze plasma lactate levels. Plasma MDA levels were analyzed using a BIOXYTECH LPO-586 kit (Oxis International, Beverly Hills, CA, USA). A 200-μL aliquot of plasma or standard was mixed with 640 μL of diluted N-methyl-2-phenylindole. Thereafter, 150 μL of concentrated hydrochloric acid was added, mixed, and incubated at 45 °C (60 min). After cooling, the absorbance values of the standards and samples were read at 586 nm using a spectrophotometer (HP 8452A; Hewlett-Packard, Palo Alto, CA, USA). Plasma SOD activity was analyzed using a tetrazolium-based kit (IBL, Hamburg, Germany). A 200 μL sample of the diluted radical detector and 10 μL plasma sample were added to prepared standard wells. Next, 20 μL of diluted xanthine oxidase was divided into the wells, mixed for several seconds, and incubated at 18–20 °C for 20 min. The absorbance was measured at 450 nm using a spectrophotometer (Tecan Sunrise; TECAN GmbH, Salzburg, Austria).

#### 2.4.3. Lymphocyte DNA Damage Analysis

Lymphocyte DNA damage was measured using the Comet assay following the previously published protocols by Singh et al. [28] and Green et al. [29]. Extracted whole blood (120 μL) and phosphate-buffered saline (PBS) (900 μL) were mixed and centrifuged at 1450 rpm for 4 min in lymphocyte separation solution (Histopaque-1077, Sigma Chemical Co., St. Louis, MO, USA). The isolated lymphocytes were then washed with PBS, centrifuged further, and mounted on slides using agarose followed by immersion in lysis solution (pH 10, 2.5 M NaCl, 100 mM Na2EDTA, 10 mM Tris, 1% sodium sarcosinate, 1% Triton X-100, and 10% DMSO) at 4 °C for 1 h. The slides were placed in electrophoresis buffer for unwinding, run in a horizontal gel electrophoresis tank, and the results were observed using fluorescence microscopy after staining the slides with ethidium bromide. Images were evaluated by measuring the percentage of DNA (tail DNA percentage [TD]), the distance of DNA fragment migration (tail length [TL]), and tail moment (TM), which was calculated by multiplying TD and TL values for 50 cells per slide using Comet 5.0 image analyzer software (Kinetic Imaging, Liverpool, UK).

### 2.5. Statistical Analysis

Mean and standard deviation of all variables were determined using the SPSS 25.0 statistical package (IBM Corp., Armonk, NY, USA). Two-way repeated measured analysis of variance (ANOVA) was conducted to examine the difference between time and group of each dependent variable. One-way ANOVA was conducted for the interaction and to determine statistical significance, which was set at *p* < 0.05.

## 3. Results

### 3.1. Plasma Lactate Levels

The change in plasma lactate levels based on aerobic fitness levels and the C242T polymorphism after exhaustive exercise is shown in Table 3. All groups displayed a significant increase in plasma lactate levels at IAE rather than at IBE and significant reduction at 30 MAE (*p* < 0.05). However, no significant difference was observed between the groups (*p* > 0.05).

### 3.2. Plasma MDA Levels and SOD Activity

The change in plasma MDA level based on aerobic fitness levels and the C242T polymorphism after exhaustive exercise is shown in Table 3. All groups displayed a significant increase in plasma MDA levels at IAE rather than at IBE, and the CC + HF and T + HF groups presented a significant reduction at 30 MAE (*p* < 0.05). Furthermore, plasma MDA levels were significantly higher in the CC + MF group than in the T + HF group at IAE (*p* < 0.05). Plasma SOD activity was significantly increased in all groups at IAE rather than at IBE and significantly decreased at 30 MAE (*p* < 0.05). However, no significant difference was observed between the groups (*p* > 0.05).

### 3.3. Lymphocyte DNA Damage

#### 3.3.1. Change in TD

The change in TD based on aerobic fitness levels and the C242T polymorphism after exhaustive exercise is shown in Table 4. TD was significantly increased in all groups at IAE rather than at IBE and significantly decreased at 30 MAE (*p* < 0.05). However, no significant difference was observed between the groups (*p* > 0.05).

#### 3.3.2. Change in TL

The change in TL based on the aerobic fitness level and the C242T polymorphism after exhaustive exercise is shown in Table 4. All groups showed a significant increase in TL at IAE rather than at IBE and significantly reduced at 30 MAE (*p* < 0.05). Moreover, the TL was significantly higher in the CC + MF group than in the T + HF group at IAE (*p* < 0.05), and both CC + MF and CC + HF groups exhibited significantly higher values than the T + HF group at 30 MAE (*p* < 0.05).

#### 3.3.3. Change in TM

The change in TM based on aerobic fitness levels and the C242T polymorphism after exhaustive exercise is shown in Table 4. TM was significantly higher in all groups at the IAE rather than at the IBE and significantly decreased at 30 MAE (*p* < 0.05). Furthermore, TM was significantly higher in the T + MF group than in the T + HF group at IAE (*p* < 0.05), and TM of the CC + MF group was significantly higher than that of the CC + HF and T + HF groups at IAE and 30 MAE (*p* < 0.05).

## 4. Discussion

NOXs constitute an important source of active oxygen along with mitochondria. Mammalian NOXs, including those of humans, comprise NOX1–5 and dual oxidases 1 and 2. These are composed of gp91^phox^ (cytochrome b558 heavy chain), primarily located within the cell membrane, and subdivided according to structural differences, including p22^phox^, p47^phox^, p67^phox^, p40^phox^, and the small GTP-binding protein Rac [30,31]. Among these, the gene encoding p22^phox^ is located on chromosome 16q24 and harbors the C242T polymorphism, through which histidine (His) is substituted with tyrosine (Tyr) at the 72nd codon, showing three associated genotypes (CC, CT, and TT) [16,17]. Several studies have reported differences in the distribution of alleles in the C242T polymorphism according to race (ethnicity). For example, Hashad et al. [19] analyzed the C242T polymorphism in an Egyptian population, reporting a 27.7% frequency for the CC genotype, 71.3% for the CT genotype, and 1% for the TT genotype. De Caterina et al. [32] reported frequencies of 36.4%, 47.7%, and 15.9%, respectively, among 1864 Italian individuals, indicating that the CT genotype displayed the highest distribution. Alternatively, Yamada et al. [33] analyzed the C242T polymorphism in 1074 Japanese subjects and reported that 816 (76.0%) harbored the CC, 242 (22.5%) harbored CT, and 16 (1.5%) reported TT genotypes. Similarly, an analysis of a Chinese population [34] revealed genotype frequencies of 87.2%, 12.2%, and 0.6%, respectively. Our results are consistent with those of other studies, including genotype frequencies at 81.8%, 16.4%, and 1.8%, for CC, CT, and TT genotypes, respectively, suggesting that the distribution of the CC genotype is highest among Asians.

Lactate as an energy source is resynthesized to glucose in the liver and muscle or re-converted to pyruvate and constitutes an indispensable resource that is produced to satisfy rapid energy requirements. Because lactate is produced at high levels during continuous high-intensity exercise, it can be utilized as a fatigue marker to determine exercise intensity [35,36]. In particular, plasma lactate levels herein were significantly higher at IAE than at IAB and significantly lower at 30 MAE. However, no significant differences were observed among the groups, suggesting that one-bout exercise can induce lactate accumulation regardless of aerobic fitness level and C242T polymorphism status, as all subjects performed treadmill running at the exercise intensity corresponding to 85% VO_2_max. In particular, plasma lactate levels were increased as pyruvate production during glycolysis exceeded the maximum oxidizable amount in the mitochondria, with the increased pyruvate from lactate dehydrogenase catalysis consequently being converted to lactate. Concurrently, Ament and Verkerke [37] reported that rapid accumulation of blood lactate is generally observed when the exercise intensity increases and the switch from aerobic to anaerobic exercise metabolism occurs, which can be induced at 50% and 80% VO_2_max in both untrained and well-trained subjects relative to exercise intensity.

The *CYBA* C242T polymorphism has been studied in patients with OS-related disorders, including cardiovascular disease, with the presence of the T allele among CC, CT, and TT genotypes, which are associated with lower superoxide production [12,13]. In comparison, this study analyzed plasma MDA levels and SOD activity to examine redox state biomarkers due to exhaustive aerobic fitness levels and the C242T polymorphism. We observed a significant increase in all groups in MDA levels at IAE, whereas only the two HF groups (CC + HF and T + HF) exhibited significant reductions at 30 MAE. Furthermore, these levels were significantly higher in the CC + MF group than in the T + HF group at IAE. These results suggested that high aerobic fitness and the presence of the T allele created an advantage compared to lower aerobic fitness and the CC genotype in alleviating OS levels, which increased after exhaustive one-bout exercise, thus supporting the previous findings that the T allele of the C242T polymorphism is associated with lower superoxide production than the CC genotype [18,19,20]. Moreover, MDA, 8-OHdG, and myeloperoxidase (MPO) are blood markers of acute exercise-induced OS levels [38,39,40]. Izzicupo et al. [41] conducted maximal stress tests using the Bruce protocol investigating the C242T polymorphism in 97 healthy long-distance runners. They reported that the T allele (CT/TT) is potentially associated with significantly lower MPO levels after exercise than the CC genotype and that T carriers are characterized by a significantly lower release of MPO [41].

Alternatively, the body has elaborate antioxidant defense systems to neutralize free radicals and ROS, with antioxidant enzymes, including SOD, catalase, and glutathione peroxidase [40]. SOD reportedly functions as an important antioxidant marker as it constitutes the first defense mechanism against superoxide radicals and is critical for converting superoxide radicals to H_2_O_2_ and H_2_O [40,42]. In the present study, all groups displayed a significant increase in SOD activity at IAE and a significant reduction at 30 MAE. However, no significant differences between groups were observed, supporting previous studies that reported increased blood SOD activity after exercise [43,44] and that the activation of the antioxidant system increases to protect the body from oxidant damage due to one-bout exhaustive exercise. Specifically, Shin et al. [43] reported an increase in blood SOD activity after one-bout exercise that consumed 400 kcal at 60 and 80% of VO_2_max. Furthermore, Roh et al. [44] reported significantly increased blood SOD activities regardless of aerobic fitness level (54.3 ± 3.8 vs. 41.8 ± 6.8 mL/kg/min) after 20 min of treadmill running at 85% of VO_2_max. However, plasma SOD activity, which differs from MDA, did not display a significant difference based on the C242T polymorphism status, suggesting that the T allele was beneficial for lowering OS levels through lower superoxide production but was not directly involved in antioxidant capacity. The findings of Izzicupo et al. [41] support this conclusion, since they reported no significant difference in the total antioxidant capacity among the alleles in the C242T polymorphism, even though MPO levels were significantly lower in subjects harboring the T allele after single-bout exercise.

Despite mechanisms to minimize oxidative damage from free radicals and ROS, redox imbalance owing to a higher than necessary antioxidant capacity of the cell can result in damage to biomolecules, including DNA, and cause variations at the genome and chromosome level if the damaged DNA is not restored, thus potentially inducing modifications in gene and protein activation to initiate various diseases [45,46]. The current study examined lymphocyte DNA damage (TD, TL, and TM) through the Comet assay to investigate the effect of exhaustive one-bout exercise on oxidative DNA damage based on aerobic fitness level and C242T polymorphism. Our results indicated a significant increase in all three markers at IAE, despite a significant reduction at 30 MAE. These results supported the previous findings that one-bout exercise induces temporary lymphocyte DNA damage [47,48], which we considered to be mediated primarily by the exercise-induced OS. In comparison, the results of treadmill running at 75% of the heart rate reserve in the study by Roh et al. [47] revealed that all DNA damage markers including TD, TL, and TM significantly increase immediately after exercise. Moreover, Kim et al. [48] reported significant increases in the TD, TL, and TM after a triathlon. Furthermore, our results show that the TL was significantly higher in the CC + MF group than in the T + HF group at IAE, and significantly higher in the CC + MF and CC + HF groups than in the T + HF group at 30 MAE. Furthermore, the TM was significantly higher in the T + MF group than in the T + HF group at IAE and significantly higher in the CC + MF group than in the CC + HF and T + HF groups at IAE and 30 MAE. These results further indicate that high aerobic fitness and the presence of the T allele were beneficial for the alleviation of OS levels that were increased through exhaustive one-bout exercise.

Consistent with previous studies [18,19,20] indicating that the presence of the T allele is associated with a lower superoxide production than the CC genotype, this study revealed significantly lower plasma MDA levels, a biomarker for OS levels, in the T + HF group than in the CC + MF group immediately after exercise. Similarly, Izzicupo et al. [41] reported significantly lower MPO levels after high-intensity one-bout exercise in subjects harboring the T allele (CT/TT) than those harboring the CC genotype. Moreover, Mota et al. [49] examined the degree of DNA damage by dividing aerobic fitness levels (high or low fitness) according to age and reported that aerobic fitness is potentially associated with DNA damage because only the low fitness group exhibited DNA strand breaks. Furthermore, Moreno-Villanueva et al. [24] analyzed lymphocyte DNA damage after single-bout exercise in trained (VO_2_max > 55 mL/kg/min) and untrained (VO_2_max < 45 mL/kg/min) subjects and reported that only the latter exhibited DNA damage, whereas trained subjects rapidly repaired the damaged DNA, thus supporting the present results.

Notably, OS-related variables, such as those associated with the incidence of cardiovascular disease, may differ with the CC genotype and T allele (CT/TT) of the C242T polymorphism [18,19,20]. In addition, the present study demonstrated that the presence of the CC genotype vs. T allele (CT/TT) could affect exercise-induced OS and DNA damage in healthy young adults. However, other studies have reported no significant OS difference between the CC genotype and T allele (CT/TT) carriers [16,50], raising the possibility that the effects do not differ among races, making the results of our study challenging to generalize. Moreover, this study has several limitations. First, only a small number of subjects were recruited from a single center and region; a larger number of subjects should thus be evaluated in future studies. Second, the amount of physical activity of the subjects before testing was not examined even though physical activity can affect body oxidant/antioxidant balance. Third, the levels of secreted sex hormones, including estrogen, in 20–30-year-old healthy adults are closely associated with OS; hence, future studies are required to examine OS levels in different sexes. Fourth, as regular exercise training constitutes a primary method to alleviate OS levels, future studies should examine the effects of regular training based on the C242T polymorphism.

## 5. Conclusions

In conclusion, this study shows that, via the studied samples, single-bout exhaustive exercise induces the accumulation of a peripheral fatigue marker, temporary redox imbalance, and oxidative DNA damage. Furthermore, the present results suggest that high aerobic fitness and the presence of the T allele potentially alleviate exercise-induced redox imbalance and DNA damage while simultaneously facilitating rapid OS alleviation and restoration of damaged DNA during recovery after exercise.

## Figures and Tables

**Table 1:** Genotype and allele frequencies of the C242T polymorphism.

	Men (*n* = 110)	Women (*n* = 110)	Total (*n* = 220)
**Genotype, *n* (%)**			
CC	89 (80.91)	91 (82.73)	180 (81.82)
CT	19 (17.27)	17 (15.45)	36 (16.36)
TT	2 (1.82)	2 (1.82)	4 (1.82)
**Allele Frequency**			
C	0.81	0.83	0.82
T	0.19	0.17	0.18

**Table 2:** Subject characteristics.

Variable	CC + HF (*n* = 8)	CC + MF (*n* = 8)	T + HF (*n* = 8)	T + MF (*n* = 8)	*p* ^#^
Sex (men/women)	4/4	4/4	4/4	4/4	
Age (years)	22.25 ± 2.31	23.38 ± 3.20	22.50 ± 2.67	22.25 ± 2.31	0.808
Height (cm)	170.55 ± 11.17	168.83 ± 6.02	172.75 ± 10.60	169.30 ± 7.55	0.827
Weight (kg)	62.16 ± 10.76	63.15 ± 9.87	63.41 ± 10.87	65.00 ± 9.83	0.957
BMI (kg/m^2^)	21.21 ± 1.50	22.04 ± 2.28	21.12 ± 1.72	22.61 ± 2.47	0.425
Body fat (%)	18.51 ± 7.14	20.38 ± 7.09	19.86 ± 7.96	21.78 ± 5.66	0.828
VO_2_max (mL/kg/min)	49.81 ± 7.16 *	41.48 ± 4.22	49.96 ± 7.47 *	40.93 ± 3.94	0.003
Exercise time to exhaustion (min)	36.75 ± 5.28	33.03 ± 5.03	38.07 ± 7.13	33.79 ± 6.08	0.295

Values represent the means ± standard deviation. BMI: body mass index; CC + HF: high fitness with the CC genotype; CC + MF: mid-range fitness with the CC genotype; T + HF: high fitness with the T allele; CC + MF: mid-range fitness with the T allele. * CC + HF and T + HF are significantly higher than CC + MF and T + MF (*p* < 0.05); ^#^ determined using one-way analysis of variance.

**Table 3:** Change of plasma lactate, MDA levels, and SOD activities according to aerobic fitness level and C242T polymorphism status after exhaustive exercise.

Variable	Group	IBE	IAE	30 MAE
Lactate (mmol/L)	CC + HF (*n* = 8)	1.13 ± 0.26	6.94 ± 2.11 *	2.68 ± 1.14
	CC + MF (*n* = 8)	1.50 ± 0.51	8.91 ± 2.33 *	3.01 ± 1.23
	T + HF (*n* = 8)	1.08 ± 0.24	8.21 ± 2.83 *	3.04 ± 1.26
	T + MF (*n* = 8)	1.35 ± 0.40	8.28 ± 1.93 *	3.06 ± 1.44
MDA (mmol/mL)	CC + HF (*n* = 8)	5.27 ± 0.81	6.07 ± 1.18 *	5.54 ± 0.74
	CC + MF (*n* = 8)	5,02 ± 1.01	7.81 ± 1.35 ^#,a^	5.94 ± 1.20
	T + HF (*n* = 8)	5.06 ± 0.72	5.97 ± 0.85 *	5.37 ± 0.75
	T + MF (*n* = 8)	5.02 ± 1.23	7.51 ± 1.73 ^#^	5.82 ± 1.38
SOD (U/mL)	CC + HF (*n* = 8)	3.10 ± 0.46	3.93 ± 0.65 *	3.75 ± 0.59
	CC + MF (*n* = 8)	3.29 ± 0.41	4.43 ± 1.15 *	3.97 ± 0.99
	T + HF (*n* = 8)	3.12 ± 0.43	3.64 ± 0.36 *	3.28 ± 0.38
	T + MF (*n* = 8)	3.20 ± 0.47	3.96 ± 0.47 *	3.58 ± 0.34

Values represent the means ± standard deviation. MDA: malondialdehyde; SOD: superoxide dismutase; IBE: immediately before exercise; IAE: immediately after exercise; 30 MAE: 30 min after exercise; CC + HF: high fitness with the CC genotype; CC + MF: mid-range fitness with the CC genotype; T + HF: high fitness with the T allele; CC + MF: mid-range fitness with the T allele. * Significant difference with IBE and 30 MAE (*p* < 0.05); ^#^ Significant difference with IBE (*p* < 0.05). ^a^ CC + MF is significantly higher than T + HF (*p* < 0.05).

**Table 4:** Change of lymphocyte DNA damage according to aerobic fitness level and C242T polymorphism status after exhaustive exercise.

Variable	Group	IBE	IAE	30 MAE
TD (%)	CC + HF (*n* = 8)	22.02 ± 5.81	37.60 ± 3.90 *	27.85 ± 4.07
	CC + MF (*n* = 8)	25.20 ± 5.74	38.84 ± 5.38 *	29.27 ± 3.79
	T + HF (*n* = 8)	19.55 ± 6.66	33.27 ± 6.49 *	26.67 ± 5.58
	T + MF (*n* = 8)	23.98 ± 3.95	36.26 ± 8.83 *	27.96 ± 6.94
TL (μm)	CC + HF (*n* = 8)	64.69 ± 14.18	94.95 ± 7.63 *	78.66 ± 8.86^b^
	CC + MF (*n* = 8)	65.67 ± 6.82	103.92 ± 10.99 *^,a^	78.87 ± 4.42 ^b^
	T + HF (*n* = 8)	53.86 ± 9.36	83.91 ± 11.84 *	63.99 ± 7.49
	T + MF (*n* = 8)	66.91 ± 7.18	99.13 ± 23.15 *	75.39 ± 13.90
TM	CC + HF (*n* = 8)	11.27 ± 3.15	23.10 ± 4.13 *	18.32 ± 3.53
	CC + MF (*n* = 8)	14.46 ± 2.04	31.40 ± 3.19 *^,c^	25.43 ± 5.69 ^c^
	T + HF (*n* = 8)	12.15 ± 3.62	21.83 ± 3.67 *	16.65 ± 5.02
	T + MF (*n* = 8)	14.49 ± 2.78	28.05 ± 3.79 *^,d^	20.77 ± 4.03

Values represent the means ± standard deviation. TD: tail DNA percentage; TL: tail length; TM: tail moment; IBE: immediately before exercise; IAE: immediately after exercise; 30 MAE: 30 min after exercise; CC + HF: high fitness with the CC genotype; CC + MF: mid-range fitness with the CC genotype; T + HF: high fitness with the T allele; CC + MF: mid-range fitness with the T allele; * Significant difference to with IBE and 30 MAE (*p* < 0.05); ^a^ CC + MF is significantly higher than T + HF (*p* < 0.05); ^b^ CC + HF and CC + MF are significantly higher than T + HF (*p* < 0.05); ^c^ CC + MF is significantly higher than CC + HF and T + HF (*p* < 0.05); ^d^ T + MF is significantly higher than T + HF (*p* < 0.05).
